# Distributed Egocentric Betweenness Measure as a Vehicle Selection Mechanism in VANETs: A Performance Evaluation Study

**DOI:** 10.3390/s18082731

**Published:** 2018-08-20

**Authors:** Ademar T. Akabane, Roger Immich, Richard W. Pazzi, Edmundo R. M. Madeira, Leandro A. Villas

**Affiliations:** 1 Institute of Computing (IC), University of Campinas (UNICAMP), 1251 Albert Einstein Av., Campinas, SP 13083-970, Brazil; immich@gmail.com (R.I.); edmundo@ic.unicamp.br (E.R.M.M.); leandro@ic.unicamp.br (L.A.V.); 2 Faculty of Business and Information Technology (FBIT), University of Ontario Institute of Technology (UOIT), 2000 Simcoe St N, Oshawa, ON L1H 7K4, Canada; richard.pazzi@uoit.ca

**Keywords:** ego-networks, sociocentric centrality measures, egocentric centrality measures, egocentric betweenness measure, vehicular ad hoc networks (VANETs), social network analysis (SNA)

## Abstract

In the traditional approach for centrality measures, also known as sociocentric, a network node usually requires global knowledge of the network topology in order to evaluate its importance. Therefore, it becomes difficult to deploy such an approach in large-scale or highly dynamic networks. For this reason, another concept known as egocentric has been introduced, which analyses the social environment surrounding individuals (through the ego-network). In other words, this type of network has the benefit of using only locally available knowledge of the topology to evaluate the importance of a node. It is worth emphasizing that in this approach, each network node will have a sub-optimal accuracy. However, such accuracy may be enough for a given purpose, for instance, the vehicle selection mechanism (VSM) that is applied to find, in a distributed fashion, the best-ranked vehicles in the network after each topology change. In order to confirm that egocentric measures can be a viable alternative for implementing a VSM, in particular, a case study was carried out to validate the effectiveness and viability of that mechanism for a distributed information management system. To this end, we used the egocentric betweenness measure as a selection mechanism of the most appropriate vehicle to carry out the tasks of information aggregation and knowledge generation. Based on the analysis of the performance results, it was confirmed that a VSM is extremely useful for VANET applications, and two major contributions of this mechanism can be highlighted: (i) reduction of bandwidth consumption; and (ii) overcoming the issue of highly dynamic topologies. Another contribution of this work is a thorough study by implementing and evaluating how well egocentric betweenness performs in comparison to the sociocentric measure in VANETs. Evaluation results show that the use of the egocentric betweenness measure in highly dynamic topologies has demonstrated a high degree of similarity compared to the sociocentric approach.

## 1. Introduction

Centrality is a concept widely employed in social network analysis (SNA) to classify nodes as central or, more importantly, in the network [1,2]. Several approaches have been developed to compute node centrality [2]; however, the three most commonly-used approaches in SNA are degree centrality, closeness centrality and betweenness centrality [3,4]. Although there are different centrality metrics in the literature, most of them fit into two categories such as radial and medial measures [2]. Radial measures assess information flow that originates from, or ends at, a given node. It includes degree and closeness centrality. On the other hand, medial measures assess the geodesic distance that crosses through a given node [2], which includes all variations of the betweenness centrality.

The calculation of centrality measures requires global knowledge of the network topology [5], and more often than not, this knowledge is not available. Besides, it is usually difficult to obtain this information in large-scale or highly dynamic networks. Taking this into consideration, the concept of the ego-network has attracted great attention in the scientific community. This stems from the fact that its topological analysis can be carried out locally by individual nodes without the need for global knowledge of the network [5,6,7]. Another advantage of the ego-network is the simple structure to collect data compared to collecting data from the entire network. By definition, the ego-network is a subnetwork centred on a single node, called the ego, whereas one-hop nodes are called alters [5,7]. In an ego-network, only the nodes that are directly connected to the ego belong to the subnetwork [5,7]. Based on the idea of ego-networks and the betweenness centrality measure, another perspective in SNA has emerged, named egocentric betweenness [5]. The egocentric betweenness measure has been adapted for several types of networks such as wireless sensor networks [8], delay-tolerant networks [9] and wireless mesh networks [10]. However, this measure has not been systematically investigated in vehicular ad hoc networks (VANETs), which have unique characteristics such as high mobility of nodes, short connection time and frequent network partitioning.

One of the aspects of VANETs that sets them apart from other networks is their highly dynamic topology [11]. The nodes in these networks are mainly composed of vehicles, and their mobility is restricted and imposed by traffic conditions and road pathways [12,13,14]. Moreover, the technologies commonly used in VANETs are sensing, storage, computing and wireless communication devices. In this type of network, any external interaction is done through communication links either between a pair of vehicles (vehicle-to-vehicle) or between the vehicle and roadside unit (vehicle-to-infrastructure) [12,15].

The development of services over VANETs has attracted researchers from both academia and industry due to the wide diversity of applications. They can range from vehicle traffic monitoring, system-aided navigation and cooperative collision warning, to infotainment [16,17,18,19]. Many of these services need to be aware of the local situation [16,17]. To reach this awareness, one can take advantage of either cooperative awareness message (CAM) [20] (European standard) or the basic safety message (BSM) [21] (American standard). In both standards, the messages contain information regarding vehicle status such as position, speed, direction, location coordinates and other vehicle information [22]. The process of acquiring local awareness is usually performed by broadcasting one-hop messages. As a result, each vehicle will be aware of its neighbour’s vehicles within its transmission range. The periodic exchange of one-hop messages is known as beaconing [22].

Due to the instabilities in the communication links induced by the highly dynamic topology, calculating the betweenness centrality scores in a VANET is a challenging task. On the other hand, once having identified the highest-betweenness centrality node in the network, it can be used as a facilitator node to spread the information flow [9]. This measure has been frequently applied in the design of efficient data forwarding algorithms, for instance, in wireless sensor networks [8].

A distributed approach to calculate the egocentric betweenness score was implemented and evaluated with the sociocentric metric in order to prove the feasibility of the egocentric betweenness measure in VANETs. To this end, we use a beaconing mechanism to broadcast one-hop messages about its local information. Once local information is received, each vehicle can compute its egocentric betweenness score. The main goal is to present the similarity of betweenness centrality considering two approaches: local knowledge-based (egocentric) and global knowledge-based (sociocentric). In addition, we apply the egocentric betweenness measure as a vehicle selection mechanism (VSM) to find the best-ranked vehicles in the network, for each topology change, in order to carry out the tasks of information aggregation and knowledge generation. The goal is to demonstrate the need of a VSM in distributed VANET applications. Furthermore, we want to prove that this mechanism can reduce bandwidth consumption taking into account the challenges of VANETs.

The proposed approach was experimentally validated by an extensive set of simulations with different traffic densities in a real urban scenario. Simulation results have shown that egocentric betweenness scores do not correspond perfectly to the sociocentric betweenness scores in high dynamic topologies. However, it is worth highlighting the results about the similarity ranking of nodes because in most solutions, the ranking of the nodes is more important than their absolute scores [8,9,10]. In addition, our results have also demonstrated that the use of the egocentric betweenness measure as a VSM is very useful to reduce bandwidth consumption in a distributed VANET application.

To the best of our knowledge, this is one of the first experimental studies that applies egocentric betweenness centrality in VANETs, in addition to demonstrating one of the applications in VANETs. The contributions of this paper can be summarized as follows:

- The proposal of a distributed approach to compute egocentric betweenness scores over VANETs, in which vehicles only use local knowledge of the network topology;
- The experimental evidence that our proposed approach is scalable to a large number of vehicles and can handle high mobility of vehicles;
- A method to characterize the importance of a node in highly dynamic networks using the egocentric betweenness measure;
- Experiment results demonstrate that the use of the egocentric betweenness measure can be a viable option as a VSM in highly dynamic networks.

The remainder of this paper is organized as follows. Section 2 provides a brief review of the related works from the literature that use centrality measures in several research areas, besides works that we used in our study case. Section 3 details sociocentric and egocentric centrality measures. Our egocentric betweenness measure study in VANETs is described in Section 4. Some numerical results and comparisons between sociocentric and egocentric are presented in Section 5. Section 6 describes a case study that assesses the egocentric betweenness measure as a VSM for the knowledge generation about traffic congestion. Finally, Section 7 concludes this work and looks toward future work.

## 2. Related Work

We survey the works that use the egocentric betweenness measure in different areas, such as wireless sensor networks [8], mobile ad hoc networks [9] and wireless mesh networks [10] in Section 2.1. Each distinct area has had to deal with several critical issues related to their own characteristics. In VANETs, the critical issue is to deal with a highly dynamic topology. Section 2.2 provides works that we used in comparison with our experimental results (Section 6).

### 2.1. Egocentric Betweenness Measure Used in Different Areas

Cuzzocrea et al. [8] investigated the problem of the quality of service (QoS)-based topology control over wireless sensor networks. To this end, a weighted, bidirectional topology-control algorithm named edge-betweenness centrality (EBC) was proposed. EBC selects the suitable set of neighbours in which input QoS requirements may be satisfied. The idea here is to select from the target network appropriate logical neighbours of the former nodes, i.e., a subset of neighbours that can be employed to perform application-specific procedures (for instance, message delivery) without the need to include all nodes of the network. The authors have demonstrated that this approach allows achieving a high QoS in wireless sensor networks by means of evaluating the relationships between entities of the network (i.e., edges). This provides the capability of controlling the information flow, the message delivery, the latency and the energy dissipation among nodes.

The authors of SimBetrouting [9] proposed an algorithm for forwarding data packets in disconnected delay-tolerant MANETs based on social network analysis techniques. For this purpose, they designed and implemented the routing protocol, which used two components: (i) betweenness utility, which exploits the exchange of pre-estimated egocentric betweenness centrality scores; and (ii) similarity utility, which selects the node that provides the maximum utility for carrying the message. Based on these components, SimBet chooses which node provides the maximum utility for carrying the message. Simulation results have shown that it achieves good performance comparable to epidemic routing, with low network overhead. Additionally, the authors have illustrated that the employment of the egocentric betweenness metric may prove useful in any distributed systems, where global topology knowledge is inaccessible and, especially, where the underlying networks present small-world characteristics.

Vazquez-Rodas et al. [10] proposed a protocol for topology control in wireless mesh networks to improve the energy efficiency and the battery lifetime. The proposed mechanisms chooses which devices must act as routers, forwarding the data packets received from other hand-held devices to it. In order to select the devices, centrality metrics are applied, from social network analysis, to build a topology control mechanism based on a connected dominating set. The mechanism’s implementation and evaluation have been carried out in two modes, i.e., centralized and distributed. In the centralized mode, the three most common centrality measures (degree, closeness and betweenness) were employed. In the distributed mode, the egocentric betweenness measure was applied. Through the experiment results, it was verified that the use of the centrality measures contributes to a better network performance.

### 2.2. Distributed System for Information Management and Knowledge Distribution

The work of [23] has proposed a probabilistic aggregation for knowledge generation. This approach uses a hierarchical aggregation technique called soft-state sketches. This technique is an extension of Flajolet–Martin sketches [24]. The fundamental characteristic of this approach lie in the fact that the aggregate information does not have a specific value of the monitored place, for instance, an average speed of a determined road. The aggregated information has, instead, a probabilistic value. The main benefit of this approach is the capability to combine the aggregated values, with the same context, for knowledge generation. However, this work lacks a VSM to perform knowledge generation task. Therefore, all vehicles would perform such a task, thereby generating highly redundant traffic of knowledge.

Yu et al. [25] have proposed an adaptive forwarding delay control, named catch-up, to gather aggregated local information from different sources for knowledge generation. To this goal, the forwarding speed of nearby information is dynamically adjusted. Thereby, each aggregate information can have one of the two types of adaptive delays, RUN (short) or WALK (long). The delay calculation is based on a distributed learning algorithm, in which each vehicle learns by means of local information. The main advantage of catch-up is the use of an adaptive forwarding delay for knowledge generation, as well as probabilistic aggregation. However, a disadvantage of this approach is that all vehicles can act as an information aggregator and knowledge generator, which can incur network overhead.

Another solution is the data aggregation algorithm by restricting forwarders (DARF) [26]. This algorithm concentrates mainly on the selection of the vehicles that will continue the knowledge forwarding process, which was generated in the aggregation step. In order to do that, each vehicle receives one of the two available labels (forwarder or non-forwarder) according to the neighbourhood labels. As the name says, each label defines whether the vehicles will be a forwarder, or not, of the knowledge. The vehicle will be a non-forwarder if there is a forwarding vehicle immediately in front of and behind it. One of the advantages of DARF is the broadcast suppression mechanism applied during the knowledge distribution process, which is not applied in the above-mentioned works. However, it is possible to notice that there is no VSM to aggregate local information and generate the knowledge. In this way, it allows highly redundant traffic of knowledge in the network.

All systems presented here have the same shortcoming, the absence of a VSM to carry out the tasks of information aggregation and knowledge generation. Without the selection mechanism, all the vehicles would perform such tasks, resulting in highly redundant traffic of knowledge in the network. This, consequently, will lead to high bandwidth consumption. Thus, the use of VSM contributes to improving this issue, which has not yet been addressed in the literature.

## 3. Sociocentric and Egocentric Centrality Measures

In SNA, the centrality measures indicate the importance of a node within a graph. This is performed by taking into account all connections from the node (or the ones that pass through it) to other nodes [3,9]. The importance of a node can be computed by means of centrality measures such as degree, closeness, betweenness, and among many others. SNA can be divided into two network analysis approaches: ego-network analysis (egocentric) and global network analysis (sociocentric). The former studies the relationships existing from the perspective of a participant. The latter tries to observe all relationships between the participants within the network. In this section, we will study the difference between sociocentric and egocentric centrality measures for network analysis. In Section 3.1, the most commonly-used centrality measures in sociocentric analysis will be described, while in Section 3.2 the centrality measure used in egocentric analysis will be detailed. Finally, Section 3.3 gives the complexity analysis of both measures.

### 3.1. Sociocentric Centrality Measures

Centrality measures are the most useful mathematical models developed for SNA [27]. These measures aim to understand the structural properties of social relationships. For instance, a participant with a high centrality score usually has a higher degree of influence than other participants within the network. According to the SNA, the network structure consists of an undirected graph, and its definition is presented below.

**Definition** **1.** Let G=(V,E) where V corresponds to a set of nodes (v), also called vertices or actors and E corresponds to a set of edges (e, where e∈E⊆V×V is identified by a pair of nodes), also called ties. We represent the neighbourhoods of the node $v^{\prime }$ as set of nodes v∈V reachable in r hops ($N_{r}^{v}$). Thereby, $N_{r}^{v}=\left\{v^{\prime }\in V|v^{\prime }\neq v\wedge d\left(v,v^{\prime }\right)\leq r\right\}$, where d represents the geodesic distance between nodes. Furthermore, a graph can be defined as a two-dimensional adjacency matrix A, where each element $a_{ij}$ takes a value of one if an edge connects the node i to the node j (i≠j) and zero otherwise.

Freeman’s degree, closeness and betweenness measures are the most commonly-used centrality metrics in sociocentric analysis [2,3,9]. They are briefly described below.

Degree centrality is the simplest and the most well-known measure. It assesses the number of direct ties that involve a given node, i.e., it is the number of adjacent edges [3]. A node with a high degree of centrality can be seen as popular because it has a large number of ties to others [28]. According to the work of Wasserman and Faust [1], the degree can also be considered as a measure of local centrality. Therefore, degree centrality of a given node, $p_{i}$, can be mathematically represented as:

(1) $$C_{D}\left(p_{i}\right)=\sum_{j=1}^{N}e\left(p_{i},p_{j}\right)$$

where $e(p_{i},p_{j})$ = 1 means a direct link exists between $p_{i}$ and $p_{j}$, otherwise $e(p_{i},p_{j})$ = 0.

Closeness centrality is defined by the geodesic distance *d* of a subset of nodes that are mutually connected in the network [3], i.e., it measures how close a node is in relation to all other nodes in the network. This measure can be represented as an indicator of how long information will take to be propagated from a given node to other nodes within the network [4]. Therefore, closeness centrality for a given node, $p_{i}$, can be mathematically represented as:

(2) $$C_{C}\left(p_{i}\right)=\frac{\left(N-1\right)}{\sum_{j=1}^{N}d\left(p_{i},p_{j}\right)}$$

where *N* is the number of nodes in the network and i≠j.

Betweenness centrality is usually calculated as a fraction of the geodesic distance between all node pairs that pass by a determined node [4], i.e., it is based on the idea that a node is central if it is located on the shortest path between other pairs of node sets within the network. This measure is often applied as a metric of the influence of a node on the spread of information compared to other nodes of the network [28]. Therefore, betweenness centrality for a given node, $p_{i}$, can be mathematically represented as:

(3) $$C_{B}\left(p_{i}\right)=\sum_{j=1}^{N}\sum_{k=1}^{j-1}\frac{g_{jk}\left(p_{i}\right)}{g_{jk}}$$

where $g_{jk}\left(p_{i}\right)$ represents the number of geodesic paths that pass through of node $p_{i}$ and $g_{jk}$ represents the total geodesic path between $p_{j}$ and $p_{k}$.

Freeman’s centrality measures usually require global knowledge of all network nodes and their interconnections [5,7,9]. The problem here is that this knowledge is not always accessible. Furthermore, the applicability of these measures is often difficult in large-scale networks (World Wide Web) and highly dynamic networks (VANETs). This is true because in the first one, it requires a high computational power to compute all the measures, while in the second one, the interconnection topologies change rapidly over time. For this reason, the concept of ego-networks has been introduced [5,7]. The ego-network analysis can be carried out using only local knowledge, without the need for complete knowledge of the network topology.

### 3.2. Egocentric Centrality Measures

**Figure 1:**
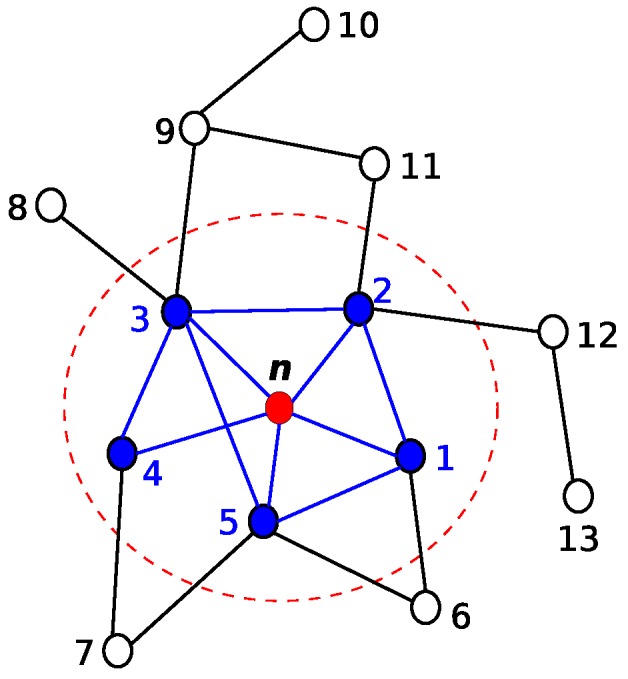
An illustration of the ego-network (local subgraph), where *n* represents the ego and the nodes (1, 2, 3, 4 and 5) denote the alters.

First of all, the definition of ego-networks is needed in order to understand the concept of egocentric centrality measures. By definition, an ego-network is a local subgraph consisting of a single node (ego) in addition to nodes that are connected to it (alters) and all the interconnection links among alters [5,6]. Figure 1 highlights a local subgraph where *n* represents ego and the one-hop neighbours (1, 2, 3, 4 and 5) denote the alters.

Inside the ego-network, the degree centrality of the nodes can be easily computed, as it is the number of direct connections of one node to its immediate neighbourhood. Because of that, it is possible to conclude that the degree centrality is similar to both egocentric and sociocentric network topologies. Incidentally, this same conclusion was reported by Wasserman and Faust [1]. On the other hand, the closeness centrality measure concerns the geodesic distances from a given node to all other nodes within the network. It is possible to notice that this measure requires the participation of all nodes involved in the network. Thereby, this measure cannot be directly applied in ego-networks, since all geodesic distances from the ego to other nodes are one-hop neighbours by definition, and this holds true because geodesic paths are no greater than two. Among the three measures presented in Section 3.1, the betweenness centrality measure is the most studied in several fields [5,29]. However, the literature lacks an investigation of this measure on VANETs.

The betweenness centrality in ego-networks will be analysed in the remainder of this section. From now on, we are going to call it the egocentric betweenness measure (EBM). The definition and how it is computed are presented below.

**Definition** **2.** Once again, let an undirected graph G=(V,E) where V corresponds to a set of nodes (v) and E corresponds to a set of edges (e, where e∈E⊆V×V is identified by a pair of nodes). The neighbourhoods of the node $v^{\prime }$ are expressed as set of nodes v∈V reachable in r hops. Let $N_{n}^{r}$ be the set of nodes that is r hops away from n (ego), i.e., $N_{n}^{r}=\left\{v^{\prime }\in V|v^{\prime }\neq n\wedge 1\leq d\left(n,v^{\prime }\right)\leq r\right\}$, where $d(n,v^{\prime })$ denotes one hop between n and $v^{\prime }$. Thereby, the first-order of node n consists of an undirected graph $G=(V_{n}^{1},E_{n}^{1})$, where the set of nodes corresponds to $V_{n}^{1}=\left\{N_{n}^{1}\cup \left\{n\right\}\right\}$ and the set of edges corresponds to $E_{n}^{1}=\left\{\left(i,j\right)\in E_{n}^{1}|i,j\in V_{n}^{1}\right\}$.

The EBM of a certain node, *n*, can be calculated by the sum of reciprocal values of the $A_{n}^{2}{\left[1-A_{n}\right]}_{i,j}$, as defined in Equation (4) [6].

(4) $$EBM_{\left(n\right)}=\sum_{\begin{array}{c} A_{n}\left(i,j\right)\neq 0,i<j \end{array}}\frac{1}{A_{n}^{2}{\left[1-A_{n}\right]}_{i,j}}$$

where $A_{n}$ depicts the adjacency matrix of the node *n*, 1 is a matrix of all ones and the matrix $A_{n}^{2}$ provides the number of geodesic distances of a length of two between node pairs *i* and *j*.

Mathematically, an adjacency matrix ($A_{k\times k}$) can represent node-to-node inter-communication links, where *k* is the number one-hop neighbours. Thereby, each element of the adjacency matrix, $a_{i,j}$, is given by:

$$a_{ij}=\left\{\begin{array}{cc} 1 & \;\mathrm{if}\;a\;\mathrm{direct}\;\mathrm{link}\;\mathrm{exists}\;\mathrm{between}\;i\;\mathrm{and}\;j\\ 0 & \;\mathrm{otherwise} \end{array}\right.$$

**Figure 2:**
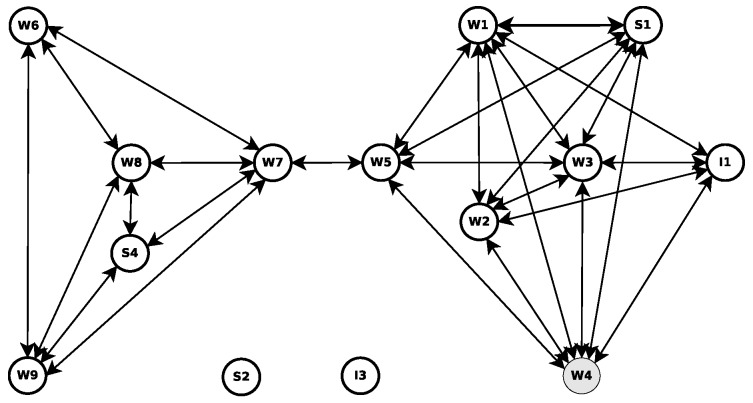
A classical graph example [5].

To demonstrate the calculation of the egocentric betweenness measure using the adjacent matrix, we employed a classical graph example [5]; see Figure 2.

Just to give one example, the egocentric betweenness score from the perspective of node W4 of Figure 2 is computed. The following adjacency matrix describes a view of all connection links between W4 (ego) and its alters, as well as the connection links between the alter pairs.

$$\;W4=\begin{array}{cc} & \begin{array}{ccccccc} \;W4 & \;I1 & \;S1 & \;W3 & W1 & W2 & W5 \end{array}\\ \begin{array}{c} W4\\ I1\\ S1\\ W3\\ W1\\ W2\\ W5 \end{array} & \left[\begin{array}{ccccccc} 0 & 1 & 1 & 1 & 1 & 1 & 1\\ \;\;1\;\; & \;\;0\;\; & \;\;0\;\; & \;\;1\;\; & \;\;1\;\; & \;\;1\;\; & \;\;0\;\;\\ 1 & 0 & 0 & 1 & 1 & 1 & 1\\ 1 & 1 & 1 & 0 & 1 & 1 & 1\\ 1 & 1 & 1 & 1 & 0 & 1 & 1\\ 1 & 1 & 1 & 1 & 1 & 0 & 0\\ 1 & 0 & 1 & 1 & 1 & 0 & 0 \end{array}\right] \end{array}$$

Since the adjacency matrix W4 is symmetric and according to Equation (4), only the non-zero values above the primary diagonal need to be analysed (i<j). In this case, the remaining entries of $W4^{2}\left[1-W4\right]$ are 4, 3 and 4, as shown in the matrix below.

$$W4^{2}\left[1-W4\right]=\begin{array}{cc} & \begin{array}{ccccccc} \;W4 & \;I1 & \;S1 & \;W3 & W1 & W2 & W5 \end{array}\\ \begin{array}{c} W4\\ I1\\ S1\\ W3\\ W1\\ W2\\ W5 \end{array} & \left[\begin{array}{ccccccc} \;*\; & \;*\; & \;*\; & \;*\; & \;*\; & \;*\; & \;*\;\\ {}* & * & 4 & * & * & * & 3\\ {}* & * & * & * & * & * & *\\ {}* & * & * & * & * & * & *\\ {}* & * & * & * & * & * & *\\ {}* & * & * & * & * & * & 4\\ {}* & * & * & * & * & * & * \end{array}\right] \end{array}$$

Therefore, the egocentric betweenness score of the ego node W4 is 0.83 (1/4+1/3+1/4). In this way, by using only the local knowledge available, each node can compute its egocentric betweenness score. Table 1 shows the scores of all nodes from the example of Figure 2, based on both betweenness centrality measures. Since egocentric betweenness is computed over the geodesic paths of the maximal length of two, the scores found in the egocentric betweenness measure are usually smaller than their sociocentric equivalents. However, an observation that is important to highlight is the similarity ranking of nodes.

The illustrative example given here was based on static networks; however, one of our major challenges is to perform the same calculation in highly dynamic network scenarios such as VANETs. In these networks, the egocentric betweenness score should be updated whenever a new communication link is established or when a communication link ceases to exist.

**Figure 3:**
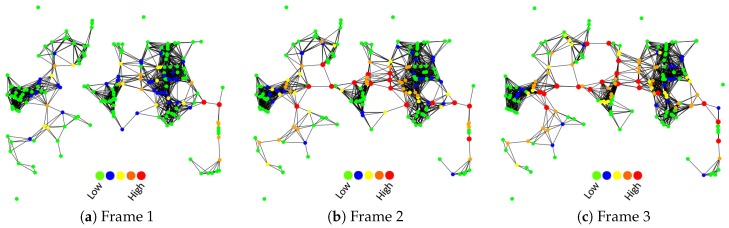
The betweenness centrality score of each node is displayed as a temporal graph, according to the evaluation scenario (traffic density of 150 vehicles/km${}^{2}$).

To exemplify how each node behaves and how the network structure can change in a highly dynamic network scenario, in relation to the betweenness centrality score, a set of footprints that describe a frame sequence (Figure 3) was illustrated; see Figure 3a–c. It shows the behaviour of the network topology (or temporal graphs) through a heat map set of our experiment scenario that will be presented later. The density is 150 vehicles/km${}^{2}$, and the transmission range is 200 m. Each node (or vehicle) is represented by a circle, and every communication link is represented by a bar. Moreover, each node can have five different colours according to the betweenness centrality score, ranging from low to high, as shown in Figure 3.

### 3.3. Complexity Analysis of the Sociocentric and Egocentric Measures

In this section, the complexity of the sociocentric and egocentric betweenness metrics is analysed. The main goal is to assess message overhead and time complexity.

For the sociocentric betweenness measure, the nodes need to collect the global network topology information before performing the calculation. A straightforward way is as follows: (i) compute the length and the number of geodesic distances between all node pairs; (ii) for each node, calculate every pair-dependency, and sum them up. Consequently, this naive algorithm will consume $\Theta (N^{3})$ time, where *N* is the number of nodes of the network. The well-known Brandes’ algorithm can be efficiently calculated in O(NM) time [30], where *N* and *M* represent the number of nodes and edges of the network, respectively. The message overhead over the entire network generally needs O(N) message copies and O(D) time steps for each node’s message, where *D* represents the network diameter [30].

For the egocentric betweenness measure, the nodes require only local network topology information to carry out the calculation. The EBM calculation demands a computation complexity equal to $O(k^{3})$ for a square matrix of k×k dimensions, where *k* is the number of alters. The message overhead over the entire ego-network topology is O(k), since each node needs to send the identification of its neighbouring nodes. Table 2 depicts the complexity analysis of the sociocentric and egocentric measures.

Since it is known that *k* is typically much smaller than *N* (k≪N), therefore the local measure approach can bring computational benefits for calculation.

## 4. Egocentric Betweenness Measure in VANETs

Based on the difficulty of calculating sociocentric centrality measures in VANETs, the egocentric betweenness measure has attracted great interest. In this section, the strategy employed in our approach to computing the egocentric betweenness scores will be addressed. Before presenting our proposed approach in Section 4.2, some assumptions are introduced in Section 4.1.

### 4.1. Assumptions

In order to compute the egocentric betweenness score, first we need to make some assumptions:

1. Each vehicle has bidirectional communication links among neighbour vehicles within transmission range. The link breaks if the distance between vehicles is greater than the transmission range;
2. All vehicles have the same transmission range;
3. The propagation model employed is two-ray interference path loss.

### 4.2. Proposed Approach

**Figure 4:**
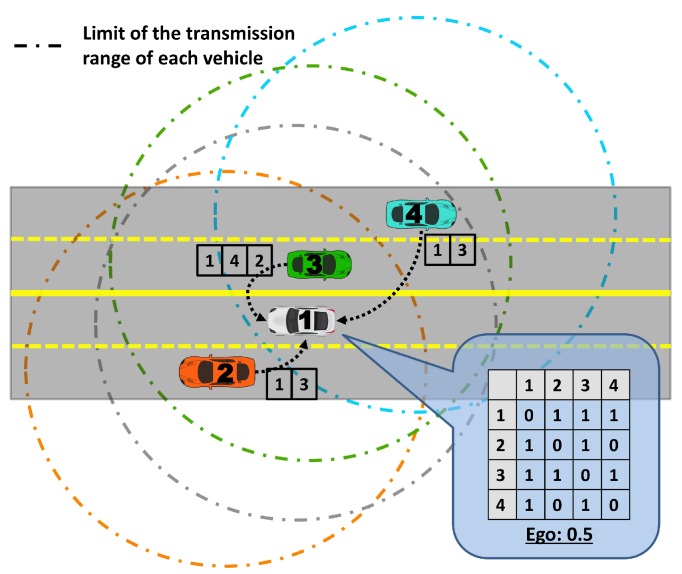
An illustrative example of the beacon packets’ exchange among the vehicles to calculate the egocentric betweenness score. In this case, the grey vehicle, labelled as 1, is doing the calculation.

Due to the high mobility of the vehicles in VANETs, getting all network topology knowledge is not an easy task. The egocentric betweenness measure is computed using only the available local knowledge; in that case, the adjacency matrix of one-hop neighbours. Each vehicle gets the local knowledge of the network topology by means of periodic beacon packets broadcast by its neighbours. The beacon transmission frequency employed was 1 Hz. Since the vehicle’s beacon packets are only useful to adjacent neighbours, the beacons received are not forwarded. Therefore, the information exchanged among vehicles is lists of neighbours, as illustrated in Figure 4. In this example with four vehicles, the grey vehicle (labelled as 1), receives the lists of neighbours of all vehicles that are currently within its transmission range (vehicles labelled as 2, 3 and 4). Once having received the lists, the vehicle constructs the adjacency matrix representation and calculates the egocentric betweenness score, according to Section 3.2. Each vehicle updates the egocentric betweenness score, whenever a new list is received.

The main steps of our proposed approach are presented in Algorithm 1. The algorithm requires the list of neighbours of all vehicles that are currently within the transmission range (represented by *L*), as input information. The output information is the current list of neighbours and the egocentric betweenness score. Upon receiving a new list of neighbours, the adjacency matrix is updated to represent a new ego-network topology (Lines 2 and 3). After the adjacency matrix is updated, the algorithm computes the egocentric betweenness score (Lines 4, 5 and 6). Thereafter, the list of neighbours is also updated (Line 7). Lastly, a beacon packet containing a current list of neighbours is broadcast (Line 8).


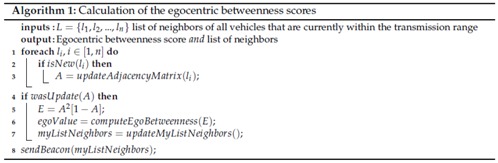


## 5. Experiments

**Figure 5:**
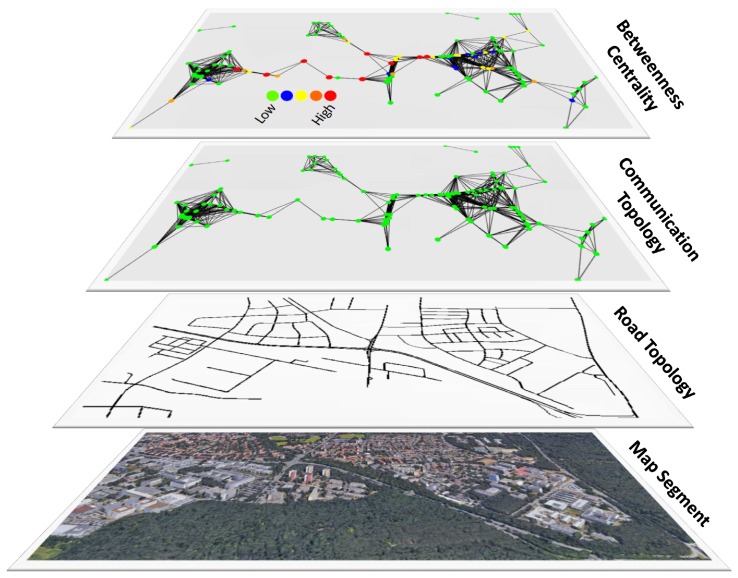
The simulation setup layers.

This work uses a distributed approach to perform the calculation of egocentric betweenness scores in vehicular networks. It consists of four stages, as depicted in Figure 5. For the sake of clarity, the figure is divided into four different layers (in a bottom-up fashion). The bottom layer represents the chosen map segment for the evaluation. The layer above it describes the road topology structure of that segment. The third layer shows the vehicle routes and the inter-vehicle communication produced in the simulation. Finally, the top layer depicts the egocentric betweenness calculation results. The next two sections describe the experimental settings (Section 5.1) employed in our simulations and the analysis of the simulation results (Section 5.2), respectively.

### 5.1. Simulation Setup

The experiments were carried out with the aid of three different simulators, namely OMNeT++ 5.0 [31] (event-based network simulator), SUMO 0.29.0 [32] (road traffic simulator) and Veins 4.5 [33] (vehicular network simulator, which integrates both aforementioned simulators). The physical (PHY) and medium access control (MAC) layers were implemented based on the WAVE (Wireless Access in Vehicular Environment) standard, also known as IEEE 802.11p.

As for simulation parameters, each vehicle had a transmission rate of 6 Mbps, a transmission power of 0.98 mW, a receiver sensitivity of −82 dBm and a transmission range of 200 m. Channel 178 (control channel (CCH)) was used to exchange beacon packets, thereby excluding the effects caused by channel switching between the CCH and the SCH (service channel).

**Figure 6:**
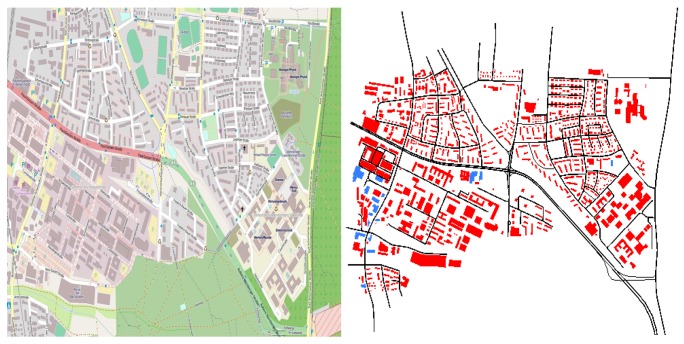
Map clipping from Erlangen, Germany. The figure on the left was imported from OSM and on the right represents the road topology used in our simulations.

In order to evaluate the applicability of the egocentric betweenness approach in vehicular networks, a real map clipping of the Erlangen area (Germany), obtained from OpenStreetMap (www.openstreetmap.org), was used (Figure 6). Meanwhile, a set of feasible vehicle routes was synthetically generated with the aid of SUMO. Vehicle mobility used the Krauss car following model [34]. Five different sets of vehicles traffic densities were generated to validate our approach (40, 60, 80, 100 and 150 vehicles/km${}^{2}$).

Finally, all experimental results of this work were executed thirty-three times on different vehicle traffic densities with a confidence interval of 95%. Table 3 summarizes the simulation parameter settings.

In order to evaluate the performance of the proposed approach, eight metrics were used and are described in detail below.

- Overhead: shows the number of beacon packets transmitted in the network by all vehicles during the simulation run;
- Beacon transmitted per vehicle: gives the number of beacon packets transmitted per each vehicle during the simulation run;
- Beacon received: displays the number of beacon packets received per vehicle during the simulation run;
- Total of lost packets: is the sum of both RxTx (receive/transmit) and SNIR (signal to noise plus interference ratio) lost packets; the first one occurs due to the busy communication channel, whereas the second one occurs due to bit errors in received packets;
- Channel busy ratio: indicates the fraction of the time in which the channel is identified as busy;
- Regression analysis: is a set of statistical processes to estimate the linear relationships between two datasets;
- Pearson correlation coefficient: expresses the strength of a linear association between two datasets;
- Window time: points out the smallest window time under which there are no changes in the egocentric betweenness.

In order to provide a better understanding of our approach, results are compared to the ones obtained from the sociocentric betweenness approach. For this purpose, a dynamic graph was generated, with the aid of the Dynamic Graph Library [35], to perform the sociocentric betweenness calculation [30]. This library requires floating car data (FCD) as the input parameter. FCD is a method applied to gather traffic knowledge. In the sociocentric approach, all the vehicle network topology knowledge was used as input.

### 5.2. Simulation Results

**Figure 7:**
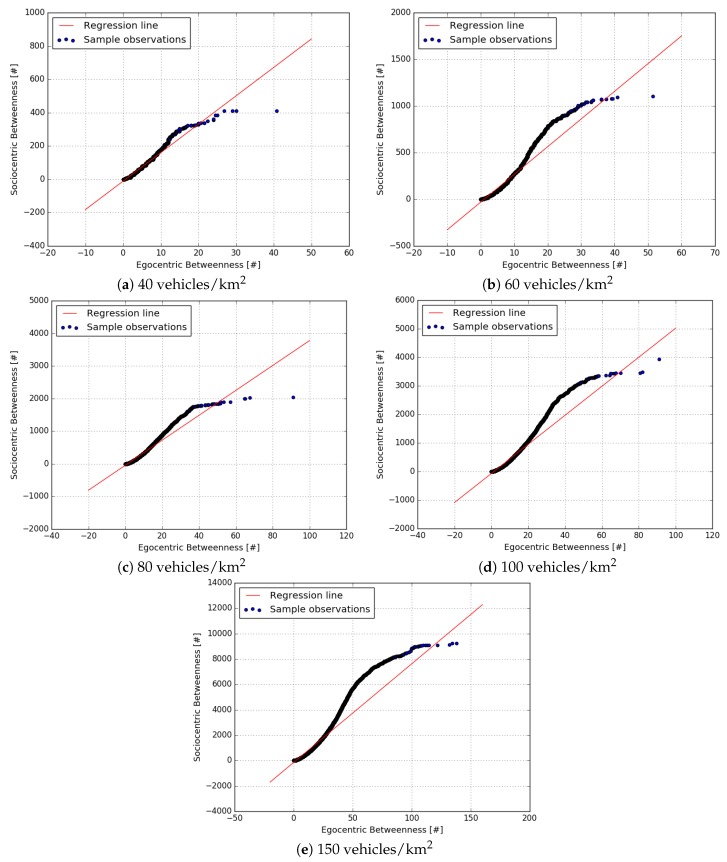
Scatterplot of sociocentric vs. egocentric betweenness for each vehicle traffic density.

The first set of experiments investigated how accurately egocentric betweenness scores correlated with the sociocentric betweenness scores in a VANET scenario; in other words, how accurate the results were when using only the local knowledge of the network topology to compute the betweenness score in highly dynamic networks, instead of using global knowledge of the topology. The results of this approach are shown in the scatter diagram set in Figure 7, which compares the two approaches for each vehicle traffic density.

A scatter plot revealed the relationships between two variables (in our case, such variables were the sociocentric and the egocentric score). The relationship between two variables is known as correlation. The higher the correlation between the two variables, the closer the sample observations will be to a straight line. If the sample observations go along a straight line (or regression line) from the origin to high x- and y-values, then the variables are assumed to have a positive correlation. Thus, it is possible to observe in Figure 7 that the egocentric and the sociocentric betweenness scores have a positive correlation.

Figure 7a–e show the scatterplots for densities of 40, 60, 80, 100 and 150 vehicles/km${}^{2}$, respectively. As can be seen in these figures, these two measures do not provide the same betweenness scores, as expected. The egocentric betweenness scores (y-axis) were smaller than the sociocentric betweenness scores (x-axis). This can be explained by the fact that in the ego-network topology, the maximal geodesic distance between nodes was two, and this limitation did not apply to the sociocentric betweenness. On the other hand, through the analysis of the figures, the egocentric and the sociocentric betweenness scores have demonstrated a high degree of similarity regarding the ranking of nodes. This similarity can be confirmed in Table 4. The table depicts the Pearson correlation coefficient (PCC) between the egocentric and the sociocentric betweenness approaches. The presented values ranged from 0.953–0.983 (where 1.0 represents a perfect linear relationship between the two datasets analysed), in all traffic densities.

Lastly, it is possible to notice that some scores lie relatively away from the regression line (red line). Even so, there is a clear positive relationship between the two betweenness measures in VANETs.

**Figure 8:**
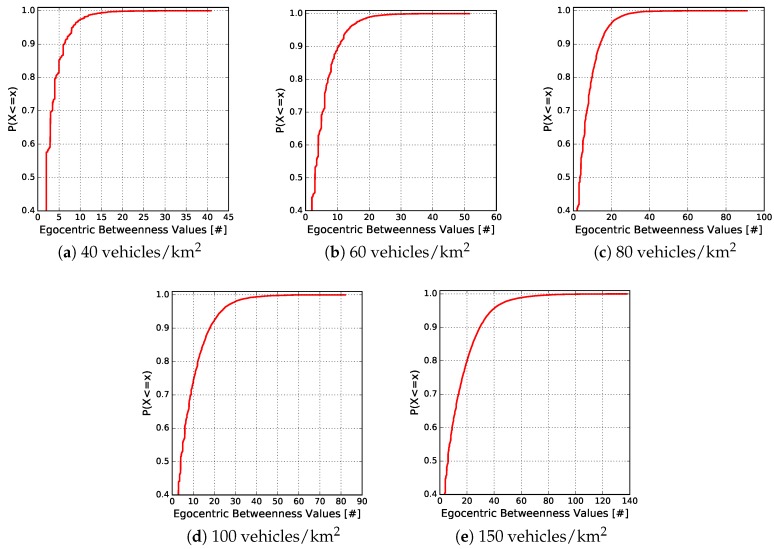
CDF of the egocentric betweenness scores in relation to the vehicle traffic densities.

**Figure 9:**
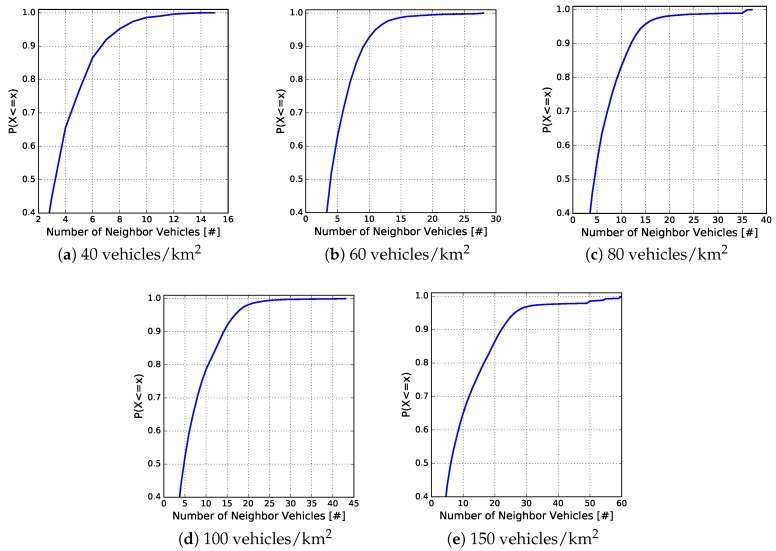
CDF of the number of one-hop neighbours in relation to the vehicle traffic densities.

Figure 8 and Figure 9 depict the cumulative distribution function (CDF), in each vehicle traffic density, of the egocentric betweenness scores and the number of one-hop neighbours, respectively. The CDF measure is an interesting way of observing the behaviour of analysed variables. As can be observed in Figure 8, the egocentric betweenness scores fluctuate in the same range as in Figure 7, according to the vehicle traffic density. Another important information is to analyse the distribution of these scores. It is possible to observe that 90% of the samples, for densities of 40, 60, 80, 100 and 150 vehicles/km${}^{2}$, were lower than 7, 11, 16, 18 and 30, respectively. In other words, these scores were close to the regression line (red line of Figure 7), i.e., 90% of the samples of the two variables had a high correlation. The same distribution analysis was performed for the number of one-hop neighbours, as shown in Figure 9. In this example, it is possible to notice that 90% of the samples, for densities of 40, 60, 80, 100 and 150 vehicles/km${}^{2}$, were lower than 7, 9, 12, 14 and 21 neighbours, respectively.

**Figure 10:**
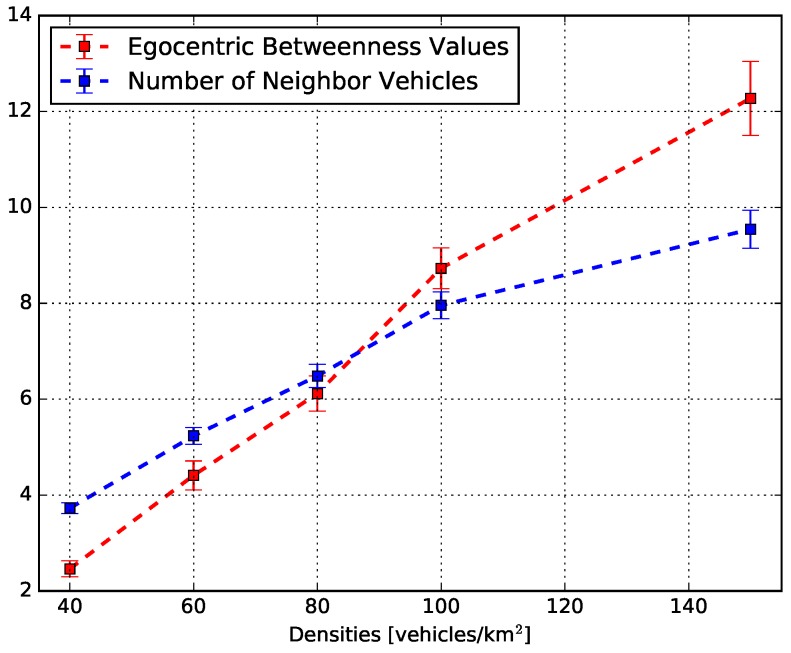
The relationship between the egocentric betweenness score and the number of one-hop neighbours.

The relationship between the egocentric betweenness scores and the number of one-hop neighbours is depicted in Figure 10. This figure shows the average egocentric betweenness score (red line) and the average number of one-hop neighbours (blue line) for all vehicle traffic densities. Therefore, it summarizes all the information presented in the two sets of Figure 8 and Figure 9. The observed behaviour of both measures is in agreement: as the traffic density increased, the number of vehicles in the vicinity and the egocentric betweenness scores also increased. For instance, in a low traffic density (40 vehicles/km${}^{2}$), the egocentric betweenness score was around 2.5, and the number of one-hop neighbours was around 3.9, on average. On the other hand, in a high traffic density (150 vehicles/km${}^{2}$), the egocentric betweenness score and the number of one-hop neighbours were around 12.2 and 9.8 on average, respectively.

**Figure 11:**
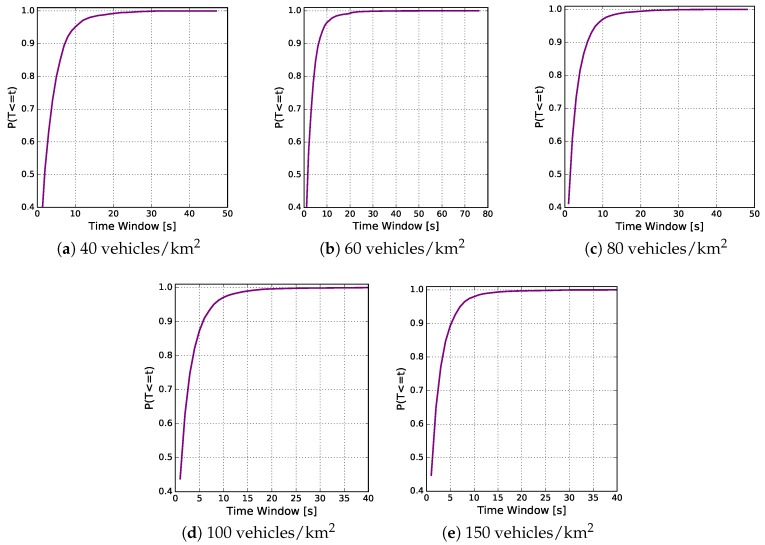
CDF of the time window duration in which there were no changes to the egocentric betweenness score in relation to the vehicle traffic densities.

Another important analysis that can be performed in the egocentric betweenness measure is the calculation of the smallest time window duration in which there were no changes to the egocentric betweenness scores in relation to the vehicle traffic densities. The CDF of the time window duration in each traffic density is shown in the Figure 11 set. In this case, it is possible to notice that 90% of the samples, for densities of 40, 60, 80, 100 and 150 vehicles/km${}^{2}$, have time window durations that were lower than 9, 8, 7, 6 and 5 s, respectively.

**Figure 12:**
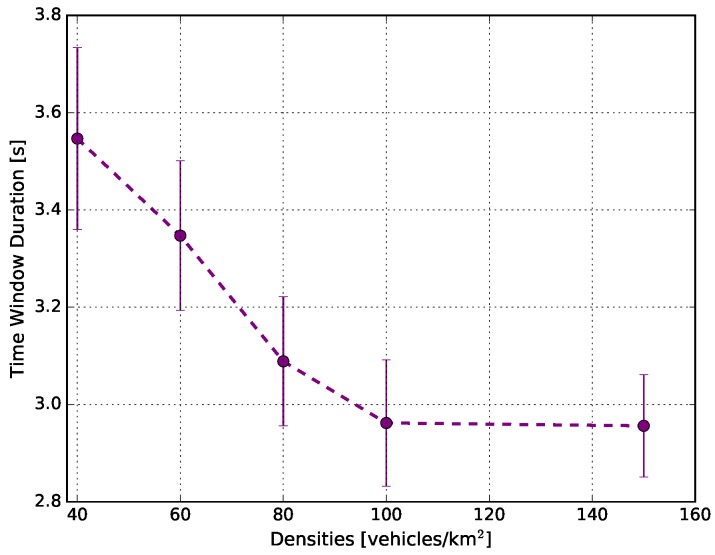
Average time window duration in which there were no changes to the egocentric betweenness scores.

Figure 12 shows the average time window duration in each traffic density. This metric is important in vehicular networks because many applications rely on a stable period of connectivity between nodes [36,37,38]. The figure shows that as the traffic increased, the average time window duration decreased, until reaching a stable plateau. For example, when the density was 40 vehicles/km${}^{2}$, the average time window was around 3.55 s. When the density increased, the average time window rapidly decreased until reaching the plateau at 2.95 s, for the cases of 100 vehicles/km${}^{2}$ and 150 vehicles/km${}^{2}$. For many distributed applications, the real-time content distribution within the area of interest was less than 2 s [37,38]. Therefore, the average time window reached into all densities of the simulations was sufficient to meet the requirements of such applications. The behaviour depicted in the picture confirmed our expectation: as traffic increased, the trend was that the list of one-hop neighbours fluctuated rapidly over time. One point worth highlighting is that the time can vary according to the scenario used, as well as the mobility model and the vehicle traffic densities applied.

**Figure 13:**
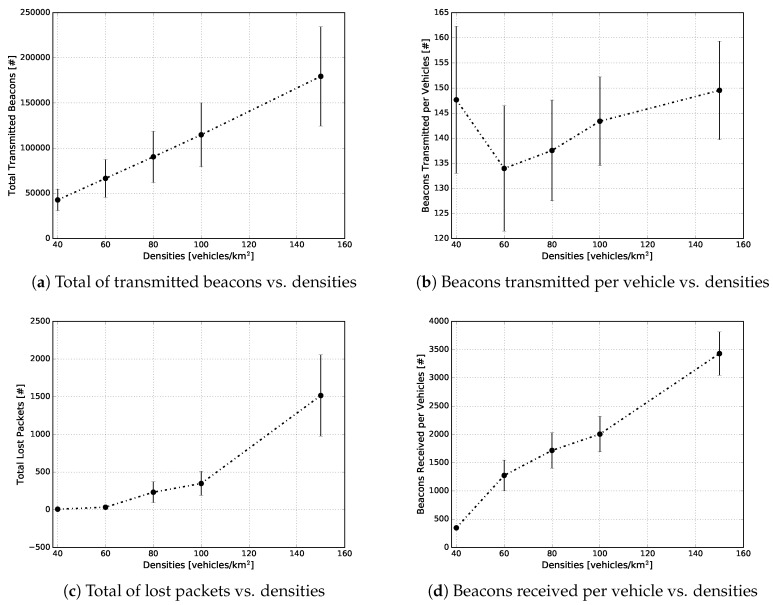
Performance evaluation of the network under different traffic densities.

The second set of experiments consisted of performing the analysis of the network traffic. This analysis is needed to demonstrate the scalability of our proposed approach, since the periodic exchange of beacon packets, to stay aware of the one-hop neighbour topology, was carried out by means of vehicle-to-vehicle communications. The experiment results of the metrics such as overhead, beacon transmitted per vehicle, beacon received and total lost packets are depicted in Figure 13. The detailed results of each one of these metrics are given below.

Figure 13a provides a macroscopic view of the total number of the beacon packets transmitted in each traffic density. For instance, in densities of 40, 60, 80, 100 and 150 vehicles/km${}^{2}$, we had on average 49,000, 70,000, 90,000, 120,000 and 180,000 transmitted beacon packets, respectively. As can be seen, the beacon overhead increased linearly as a function of the traffic density, as expected. This expectation was well founded since as the density of vehicles increased, the higher the transmission rate of beacon packets into the network would be.

**Figure 14:**
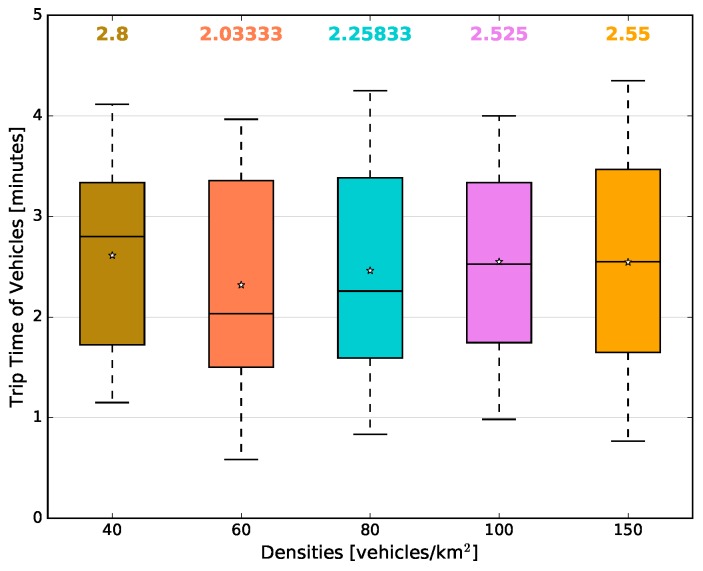
Average trip time of vehicles vs. densities.

The microscopic view is depicted in Figure 13b, which shows the average number of beacon packets transmitted by each vehicle in each traffic density. When the experimental scenario had a density of 40 vehicles/km${}^{2}$, each vehicle, on average, transmitted around 148 beacons during the simulation time; while, in the scenarios with 60 and 80 vehicles/km${}^{2}$, on average, 134 and 138 beacons were transmitted, respectively. For 100 and 150 vehicles/km${}^{2}$, there were, on average, 144 and 150 beacons transmitted by each vehicle, respectively. It is easy to see that the number of beacon packets transmitted, for each vehicle, is directly related to its trip time during the simulation time. With that in mind, Figure 14 depicts the average trip time of the vehicles during the simulation. It is possible to observe that in both of the aforementioned figures, the same behaviour appears in all the vehicle traffic densities. For example, in Figure 14, for the scenarios with 40 and 150 vehicles/km${}^{2}$, the average trip times are higher than all other evaluated scenarios, reaching 2.8 and 2.55 min, respectively. On the other hand, the scenario with 60 vehicles/km${}^{2}$ presented the lowest average (2.0 min). These behaviours are following the same pattern as in Figure 13b, as well as the confidence interval.

**Figure 15:**
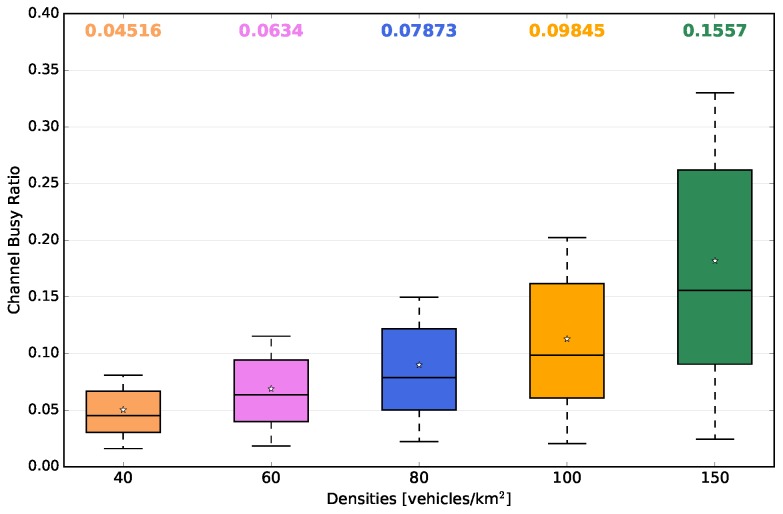
Impact on channel busy ratio vs. densities.

Figure 13c depicts the total number of beacon packets lost either by the fact that the communication channel was busy, or by errors in the received packets. As can be observed, the low densities (40 and 60 vehicles/km${}^{2}$) presented a minimum packet loss rate. As the vehicle traffic density increased up to 150 vehicles/km${}^{2}$, the total number of packets lost also increased. The observed behaviour was directly related to the channel busy ratio. Taking this into account, Figure 15 shows the average channel busy ratio for each vehicle traffic density. As the simulation time was set to 100 s, the calculation of the total busy time was nothing more than the channel busy ratio multiplied by the simulation time. In our case, for densities of 40 and 60 vehicles/km${}^{2}$, the channel was busy for the shortest time, and as the density increased, the average time also increased. Even in the density of 150 vehicles/km${}^{2}$, a maximum of 35% of channel availability was consumed. These results show that the beacon transmission frequency of 1 Hz was suitable, for this scenario, together with the mobility model applied, due to low channel utilization.

The number of beacon packets received per vehicle is depicted in Figure 13d. This metric, combined with the channel busy ratio (Figure 15), can indicate if the beacon transmission frequency is adequate or not. In the same way as the total number of beacon packets transmitted, the number of beacon packets received also increased linearly as a function of the vehicle traffic density. For instance, for densities of 40, 60, 80, 100 and 150 vehicles/km${}^{2}$, there were, on average, 480, 1300, 1700, 2000 and 3450 beacon packets received per vehicle, respectively. As mentioned before, the channel utilization in our approach was low; this confirmed, once again, that the beacon transmission frequency of 1 Hz was proper.

## 6. Egocentric Betweenness Measure as a Vehicle Selection Mechanism for Knowledge Generation about Traffic Congestion

In recent years, several intelligent transportation systems (ITS) that deal with local information management about traffic conditions have been proposed [23,25,26]. This type of system usually extracts information related to the traffic condition of a given road by processing the aggregated local information. This information is, more often than not, received from the one-hop neighbour vehicles through beacon packages. However, the above-cited systems have the same shortcoming, which is the absence of a VSM to perform the task of knowledge generation. Without any type of selection mechanism, all the vehicles are candidates to carry out such a task, resulting in highly redundant traffic of knowledge, as well as high bandwidth consumption.

In order to overcome the aforementioned limitations, we will conduct a case study to assess the impact of the EBM as a selection mechanism of the most relevant vehicle in the network for the knowledge generation process. The goal of this case study is to prove that the mechanism can reduce bandwidth consumption, taking into account the challenges of VANETs.

### 6.1. Vehicle Selection Mechanism

As mentioned before, the EBM is used to select the most relevant vehicles to carry out the task of knowledge generation. The relevance here is defined as the importance of the vehicle in relation to the information flows that pass through it.

Referring back to Table 1, it can be observed that some nodes have the same EBM score. In this particular case, three nodes have an EBM score of 0.83, two nodes have 0.25 and two nodes have 0.33. Assuming that the graph represented in Figure 2 describes the inter-vehicular communication links at a given time, as an example, if the node I1 needs to forward its aggregate local information, it, beforehand, has to select the next alter, which will be the one with the highest EBM score. As shown, I1 has three alters (W1, W3 and W4) with an EBM score of 0.83. In this case, the two-ray interference model (Equation (5)) [39] is used as the tie-breaking criterion.

(5) $$L_{TRI}\left[dB\right]=20log(4\pi \frac{d}{\lambda }|1+\Gamma \exp^{\varphi }{|}^{-1})$$

where λ is the wavelength, *d* is the Euclidean distance between two vehicles, Γ is the reflection coefficient and φ is the interfering rays. The interfering rays are given by:

(6) $$\varphi =2\pi \frac{d_{los}-d_{ref}}{\lambda },\left\{\begin{array}{c} d_{los}=\sqrt{d^{2}+{\left(h_{t}-h_{r}\right)}^{2}}\\ d_{ref}=\sqrt{d^{2}+{\left(h_{t}+h_{r}\right)}^{2}} \end{array}\right.$$

where $d_{los}$ and $d_{ref}$ correspond to the line-of-sight distance and reflected path between the transmitting and receiving antennas, respectively. $h_{t}$ and $h_{r}$ represent the transmitter and the receiver antenna heights, respectively. In this study, the same heights applied in the test bed implementation of Sommer et al.’s work were was used [39] ($h_{t}$ = $h_{r}$ = 149.5 cm). The value of λ was fixed at 0.051 m according to IEEE 802.11p [40]. Lastly, the reflection coefficient can be calculated as:

(7) $$\Gamma =\frac{\sin\theta_{i}-\sqrt{\varepsilon -\cos\theta_{i}}}{\sin\theta_{i}+\sqrt{\varepsilon -\cos\theta_{i}}},\left\{\begin{array}{c} \sin\theta_{i}=\frac{h_{t}+h_{r}}{d_{ref}}\\ \cos\theta_{i}=\frac{d}{d_{ref}} \end{array}\right.$$

where ε is the relative permittivity of the ground and θ is the angle between the ground and the reflected ray.

Following the previous example, assuming W3 was selected as the next alter, it performs the aggregation of its information along with that received; while the remaining nodes discard the received information. The information aggregation process will be carried out until reaching node W7 because, in this example, it has the highest EBM score. Once all information received has been aggregated, the W7 node is responsible for the knowledge generation. The details of this process, which includes the data aggregation technique, the procedure for knowledge generation and the broadcast suppression mechanism, will be detailed next.

### 6.2. Knowledge Generation Process and Distribution

Our proposed solution periodically shares the local information, between one-hop neighbours, through beacon packets to create the local knowledge base. In order to do that, two more pieces of information were added in the beacon package: the current EBM score and the aggregated information.

The local knowledge base is built by aggregating the local information received from the neighbourhood, as well as the calculation of the weight of the roads. Once the local knowledge base is created, the next step is to share it with the most relevant neighbour vehicle, this is performed by following the selection criterion presented in Section 6.1.

The following representation shows an example of the fusion of two aggregated values: $A_{r}:=\partial \left(A_{1},A_{2}\right)$, where ∂ is the aggregation function that has two input values ($A_{1}$ and $A_{2}$). These values are combined, resulting in a new aggregated value ($A_{r}$). As the main goal of the proposed study is the generation and distribution of knowledge about the traffic condition, the aggregation function is given as follows:

(8) $$v_{agg_{i}}^{avg}=\frac{v_{1}n_{1}+v_{2}n_{2}}{n_{1}+n_{2}}$$

where $v_{agg_{i}}^{avg}$ represents the aggregate average speed of a given road *i*. The parameters $v_{1}$ and $v_{2}$ are the two input values from *i*. $n_{i}$ indicates the amount of information that contributed to the generation of the new aggregated value. Thereby, the weight of the road *i* ($w_{i}$) is calculated as follows:

(9) $$w_{i}=\frac{v_{agg_{i}}^{avg}}{v_{spe_{i}}^{max}},\left\{\begin{array}{c} \;\;\;\;w_{i}:\;\mathrm{weight}\;\mathrm{of}\;\mathrm{road}\;i\\ v_{agg_{i}}^{avg}:\;\mathrm{aggregate}\;\mathrm{average}\;\mathrm{speed}\;\mathrm{of}\;\mathrm{road}\;i\\ v_{spe_{i}}^{max}:\;\mathrm{maximum}\;\mathrm{speed}\;\mathrm{of}\;\mathrm{road}\;i \end{array}\right.$$

After aggregating all the local information, the vehicle with the highest EBM score classifies the weight of the roads according to Table 5. The levels of service and traffic classification were based on the Highway Capacity Manual (HCM) [41].

After the classification step, if an event is identified (in our case, roads with the level of service D, E or F), a message (also known as knowledge), containing the identification of the roads in question is generated. Thereby, the knowledge distribution process in the service channel is started.

**Figure 16:**
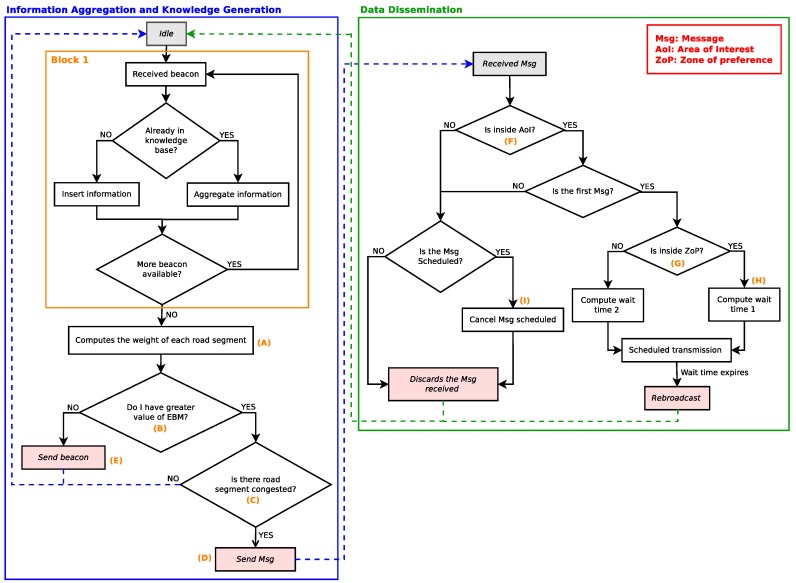
Operation flowchart of the proposed solution.

Figure 16 shows the operation flowchart of the proposed solution. The flowchart is divided into two phases. The first one is the information aggregation and knowledge generation, and the second is the data dissemination. In the first phase, every time the vehicle receives the local information, it either inserts or aggregates the local information into the local knowledge base (Block 1). In the next step, it calculates the weight of roads according to Equation (9) (Legend (A)). After this step, the vehicle with the highest EBM score (Legend (B)) classifies the weight of roads according to Table 5 (Legend (C)). During this process, if the selected vehicle detects some congested traffic flow, the knowledge is generated and distributed in the network (Legend (D)). On the other hand, if the vehicle does not have the highest EBM score, it selects the next most relevant vehicle and sends the aggregated local information to it (Legend (E)). The second phase (data dissemination), is responsible for informing vehicles that are inside an area of interest (AoI; Legend (F)) according to the application requirements. In addition, it also avoids the broadcast storm problem during the knowledge distribution process. Basically, to avoid this problem, a forwarder candidate suppresses the rebroadcast of low-priority candidates forwarders [42]. For this purpose, every time that a vehicle receives knowledge to be distributed, it checks if it is within the zone of preference [43,44] (Legend (G)), and if so, it transmits first (Legend (H)) because it has the shortest waiting time. Due to the broadcast suppression mechanism implemented (zone of preference), as soon as the neighbouring vehicles outside the zone of preference receive the same scheduled knowledge, they cancel the retransmission (Legend (I)), thereby avoiding the traffic of redundant knowledge in the network.

### 6.3. Evaluation Method

Four metrics were applied in order to evaluate the performance of the proposed solution:

- overhead: measures the total amount of transmitted messages in the network;
- collision: estimates the total number of packet collisions during message transmission;
- delay: measures the time spent in delivering the messages to vehicles;
- coverage: estimates the percentage of messages delivered to the vehicles that are within the scenario.

The simulation parameters used here are the same ones of Table 3, except the density of vehicles, which in this case ranges from 100–300 vehicles/km${}^{2}$. Moreover, AoI has been applied with a 1-km radius from the congestion point.

### 6.4. Simulation Results

**Figure 17:**
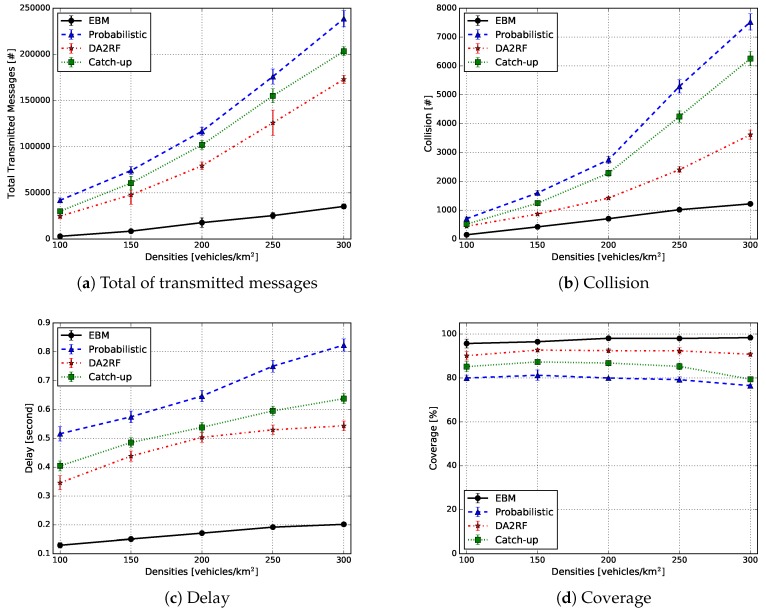
Knowledge generation results. EBM, egocentric betweenness measure.

Figure 17 shows the results of the simulations according to the metrics presented in the previous section, as a function of vehicle densities. Especially, Figure 17a displays the performance results of all solutions investigated according to the overhead metric. The probabilistic solution displays the largest number of messages transmitted because all vehicles perform the tasks of information aggregation and generation of the knowledge, resulting in highly redundant traffic of knowledge. In addition, during the knowledge distribution process, no broadcast suppression mechanism is applied, thus producing a high rate of packet collisions in the network among the analysed solutions, as shown in Figure 17b. Due to the results previously discussed, the probabilistic solution displays the lowest coverage rate, reaching an average of 80%, for all analysed densities, as shown in Figure 17d. The long delays in the delivery of knowledge, compared to the other considered solutions (Figure 17c), are also visible. It is possible to notice that there is a slight drop in the coverage rate as the vehicle density increases. This is due to the high network overhead and the high collision rate.

The other solution analysed is catch-up. It is possible to observe that the strategy of inserting an adaptive delay, in the message forwarding process, decreases the total number of messages transmitted and consequently, the collisions. This characteristic is shown in Figure 17a,b when compared to the probabilistic approach. This strategy was able to reduce, on average, 10% of both transmitted messages and packet collisions. In addition to that, catch-up increased the coverage by 5% (Figure 17d). Furthermore, in both the probabilistic approach and catch-up, there is a slight drop in the coverage rate mainly in high densities (250 and 300 vehicles/km${}^{2}$). In addition to this, catch-up still has a higher knowledge transmission rate and packet collisions compared to DA2RFand EBM. It is known that neither the probabilistic approach nor catch-up use any type of selection mechanism to choose the most relevant vehicle to perform the tasks of information aggregation and generation of knowledge. The lack of such a mechanism is translated into the higher delays for both systems when compared to DARF and EBM, as can be seen in Figure 17c.

DA2RF employs a broadcast suppression mechanism in the knowledge forwarding process. Due to this approach, it is possible to see a decrease in the total number of messages transmitted (Figure 17a). On average, DA2RF reached a reduction of 30% in comparison to the probabilistic method and 20% fewer messages when compared to catch-up. The same tendency was observed in regards to the packet collisions rate (Figure 17b). The simulation results showed a reduction of 30% and 25%, on average, compared to the probabilistic approach and catch-up, respectively. It is important to notice that DA2RF implements only the broadcast suppression mechanism and does not have any selection mechanism. Because of this, it still introduces a delay closer to the other previously analysed solutions, as depicted in Figure 17c. DA2RF’s approach improves the coverage rate by 18% and 15% when compared to the probabilistic approach and catch-up, respectively.

Finally, EBM applies the egocentric betweenness measure to perform the selection of the most relevant vehicle, which will carry out the information aggregation and knowledge generation. In addition to that, it also uses the broadcast suppression mechanism in the knowledge distribution process. This combination enables it to outperform all other solutions in all the metrics evaluated. EBM significantly reduces the total number of messages transmitted, with an average decrease of more than 85% in comparison to the probabilistic approach, as well as 80% and 70% compared to catch-up and DA2RF, respectively (Figure 17a). As a consequence of this reduction, the knowledge generated can reach a larger number of vehicles in all densities analysed, resulting in a higher coverage rate, close to 98%, on average, as shown in Figure 17d. Furthermore, the broadcast suppression mechanism implemented has helped reduce the number of packet collisions (Figure 17b). The average reduction reached more than 75%, 70% and 50% compared to the probabilistic approach, catch-up and DA2RF, respectively. At the end, the EBM system also presented the lowest average delay, among all systems analysed, being around 0.15 s (Figure 17c).

Two main lessons learned from the experimental results are as follows. The first one is that there is a need for a mechanism to select the most relevant vehicle in VANET applications. By using this type of mechanism, it is possible to make the solution more scalable. The second one refers to the egocentric betweenness measure being a viable option for the VSM in highly dynamic networks. Although our case study focused on an urban scenario, the vehicle selection mechanism is applicable to any scenario where there are vehicles on the road.

## 7. Conclusions

In this paper, we contribute to filling the gap found in the literature review and opening new avenues of research. First, a distributed approach to calculating egocentric betweenness scores, in VANETs, was presented. To this end, each vehicle regularly broadcasts one-hop messages about its local information among surrounding vehicles. The proposed approach only uses the locally available information to compute the egocentric betweenness score without the need for information of the entire network topology. A set of simulation experiments has been carried out in a real urban centre area in order to investigate the performance comparison of our egocentric approach against the traditional sociocentric approach in different vehicle traffic densities. The main contribution here is the demonstration that the egocentric approach has a greater similarity regarding the ranking of nodes in relation to the sociocentric approach. It is important to highlight those solutions that employ the egocentric betweenness measure; it is the ranking of the nodes that matters most, rather than their absolute scores. A case study of the egocentric measure as a selection mechanism of the most relevant vehicle for knowledge generation is another contribution of this work. By analysing the results, it is possible to highlight that the use of the egocentric betweenness measure is a viable option as a selection mechanism in VANETs. This is based on the fact that it overcomes two main issues: (i) the reduction of the bandwidth consumption for a distributed information management system; and (ii) the capability of dealing with the issues of highly dynamic topologies. It is worth highlighting that the egocentric betweenness measure as a vehicle selection mechanism opens new avenues of research for VANETs applications, for instance an application that has to choose the best vehicle to direct the information flow in the network.

As future works, we intend to apply an adaptive beacon rate mechanism by means of the use of the link lifetime estimation in order to reduce the exchange of beacon messages between vehicles.

## Figures and Tables

**Table 1:** Egocentric and sociocentric betweenness scores of Figure 2.

		Betweenness Centrality	
		Sociocentric	Egocentric
Nodes	W1	3.75	0.83
	W2	0.25	0.25
	W3	3.75	0.83
	W4	3.75	0.83
	W5	30.00	4.00
	W6	0.00	0.00
	W7	28.33	4.33
	W8	0.33	0.33
	W9	0.33	0.33
	S1	1.50	0.25
	S2	0.00	0.00
	S4	0.00	0.00
	I1	0.00	0.00
	I3	0.00	0.00

**Table 2:** Complexity comparison between sociocentric and egocentric measures.

Measure	Time Complexity	Message Overhead
$C_{B}\left(p_{i}\right)$	O(NM)	O(DN)
$EBM_{\left(n\right)}$	$O(k^{3})$	O(k)

**Table 3:** Simulation parameters.

Parameter	Value
Density of vehicles	40–150 vehicles/km${}^{2}$
MAC layer	802.11 p
Channel	178 (5.89 GHz)
Bandwidth	10 MHz
Transmission power	0.98 mW
Bitrate	6 Mbps
Sensitivity	−82 dBm
Transmission range	200 m
Beacon transmission frequency	1 Hz
Simulation time	350 s
Confidence interval	95%

**Table 4:** Pearson correlation coefficient (PCC) of egocentric and sociocentric betweenness.

Density (vehicles/km${}^{2}$)	PCC
40	0.983
60	0.962
80	0.971
100	0.964
150	0.953

**Table 5:** Level of service and traffic classification [41].

Level of Service	Traffic Classification	$w_{i}$
**A**	Free flow	(1.0∼0.9]
**B**	Reasonably free flow	(0.9∼0.7]
**C**	Stable flow	(0.7∼0.5]
**D**	Approaching unstable flow	(0.5∼0.4]
**E**	Unstable flow	(0.4∼0.33]
**F**	Forced or breakdown flow	(0.33∼0.0]
